# The complete mitochondrial genome of a rove beetle, *Megalinus hunanensis* Bordoni, 2013 (Coleoptera: Staphylinidae)

**DOI:** 10.1080/23802359.2021.1970648

**Published:** 2021-09-02

**Authors:** Yanpeng Cai

**Affiliations:** School of Basic Medicine, Guizhou University of Traditional Chinese Medicine, Guiyang, Guizhou, China

**Keywords:** *Megalinus hunanensis*, mitochondrial genome, Xantholinini, Staphylinidae, phylogenetic analysis

## Abstract

The complete mitogenome of rove beetle *Megalinus hunanensis* Bordoni, 2013 was determined in this study. It was 17,314 bp in length, and comprised of 13 protein-coding genes, 22 tRNA genes, two rRNA genes, and a 2511 bp A + T-rich control region. Most PCGs used the typical ATN start codon, except for *nad1*. Five genes (*cox1*, *cox2*, *cox3*, *nad4*, and *nad5*) used a single T residue as stop codon rather than the commonly used TAA or TAG. All tRNAs could be folded into the cloverleaf secondary structure. Bayesian inference phylogenetic trees built on 13 PCGs and their translated amino acid sequences from 29 rove beetle taxa showed that Staphylininae was paraphyletic with respect to Paederinae; *M. hunanensis* was a member of the tribe Xantholinini.

With about 2200 described species worldwide in more than 110 genera, Xantholinini makes the second most speciose tribe in the subfamily Staphylininae (Zhou et al. 2013; Żyła and Solodovnikov 2020). Although multiple phylogenetic analyses concerning this tribe have been conducted over the years by various authors (e.g. Cai et al. 2019; Tihelka et al. 2020; Żyła and Solodovnikov 2020), the intertribal relationships among Xantholinini and its allied tribes (such as Diochini, Maorothiini, Othiini, and Platyprosopini) are still not fully unraveled. Moreover, none of these former studies is based entirely on mitogenes and yet no complete mitogenome for such a large tribe like Xantholinini has been fully sequenced till now. The author hence presents herein the complete mitogenome of *Megalinus hunanensis* Bordoni, 2013, a rove beetle species belonging to a medium sized genus *Megalinus* Mulsant & Rey (Staphylininae, Xantholinini), which has most of its species endemic in China (Zhou et al. 2013). *Megalinus hunanensis* is a very common species in Guizhou, China, and it is characterized by the brown elytra, and the thin paler parts along the suture and the apical 1/5 of the elytra (Zhou et al. 2013).

The adults used in this study were captured in 2019, from Guiyang Huaxi District (26°19′11″ N, 106°46′18″ E, 1050 m), Guizhou, China, using haystick traps. The specimens were later put in pure alcohol before sent for sequencing. The genome sequencing was performed on Illumina HiSeq2500 platform, in Sangon Biotech (Shanghai) Co., Ltd. (Shanghai, China). The genome de novo assembly was carried out with the software MitoZ V.2.3 (Meng et al. 2019) and SPAdes V.3.14.1 (Bankevich et al. 2012). Pilon V.1.23 (Walker et al. 2014) was used for sequence correction. MITOS Web Server (http://mitos2.bioinf.uni-leipzig.de/index.py) was utilized for annotation. The remainder of the specimen tissue and the total DNA were preserved under −20 °C in the Insect Collection of Guizhou University of Traditional Chinese Medicine, Guiyang, China (Yanpeng Cai, cyp815@hotmail.com, voucher specimen: GZUTCM:001).

The full length mitogenome of *M. hunanensis* (GenBank: MW256421) was 17,314 bp in length, with double circular strands, which consisted of 13 protein-coding genes (PCGs), 22 tRNA genes, two rRNA genes, and a putative control region. The PCGs used a variety of start codons as follows. Six PCGs (*atp6*, *cob*, *cox2*, *cox3*, *nad4*, and *nad4l*) started with ATG; five (*atp8*, *cox1*, *nad2*, *nad3*, and *nad6*) with ATT; one (*nad5*) with ATA; one (*nad1*) with atypical TTG. For stop codons, four PCGs (*atp6*, *nad2*, *nad4L*, and *nad6*) used TAA; four (*atp8*, *cob*, *nad1*, and *nad3*) used TAG; while the rest five (*cox1*, *cox2*, *cox3*, *nad4*, and *nad5*) ended with a single T residue, where the stop codon was completed by the addition of a poly-A tail to the mRNA. All tRNAs could fold into the clover-leaf secondary structure, including *trnS1*, which usually lacked the DHU arm in insects. The anticodon of *trnS1* in *M. hunanensis* was UCU rather than the routinely used GCU. The overall base composition of *M. hunanensis* was AT biased, and 40.1% for A, 39.2% for T, 12.3% for C, and 8.4% for G. The non-coding control region was 2511 bp long, and strongly AT biased (AT 82.8%).

To reconstruct the phylogenetic tree, all 28 taxa (27 available from GenBank) with mitogenome longer than 13,000 pb from the two closely related subfamilies Staphylininae and Paederinae were chosen as ingroup, and a moderately close species *Tachinus subterraneus* (Tachyporinae, also from GenBank) was set as outgroup. Two datasets were used in our phylogenetic analyses: (1) DNA dataset: 13 concatenated PCG sequences; (2) AA dataset: the amino acid sequences translated from the DNA dataset. Two BI analyses were conducted via PhyloBayes-MPI V.1.8c (Lartillot et al. 2013), with CAT‐GTR substitution model used upon both datasets, because this model particularly outperformed other site-homogeneous models in robustness against systematic errors and long-branch attraction problems. The two BI trees showed the same topology (Figure 1) that was, however, unexpectedly incongruent with that of any former analyses summarized in Cai et al. (2019). Paederinae and Staphylininae failed to be recovered as monophyletic, instead, Paederinae clustered with Othiini + Xantholinini with very weak support (BPP = 0.63/0.60), rendering Staphylininae paraphyletic. *M. hunanensis* was nested within Xantholinini (BPP = 1.00/1.00), which agreed well with the classical taxonomy.

**Figure 1.**
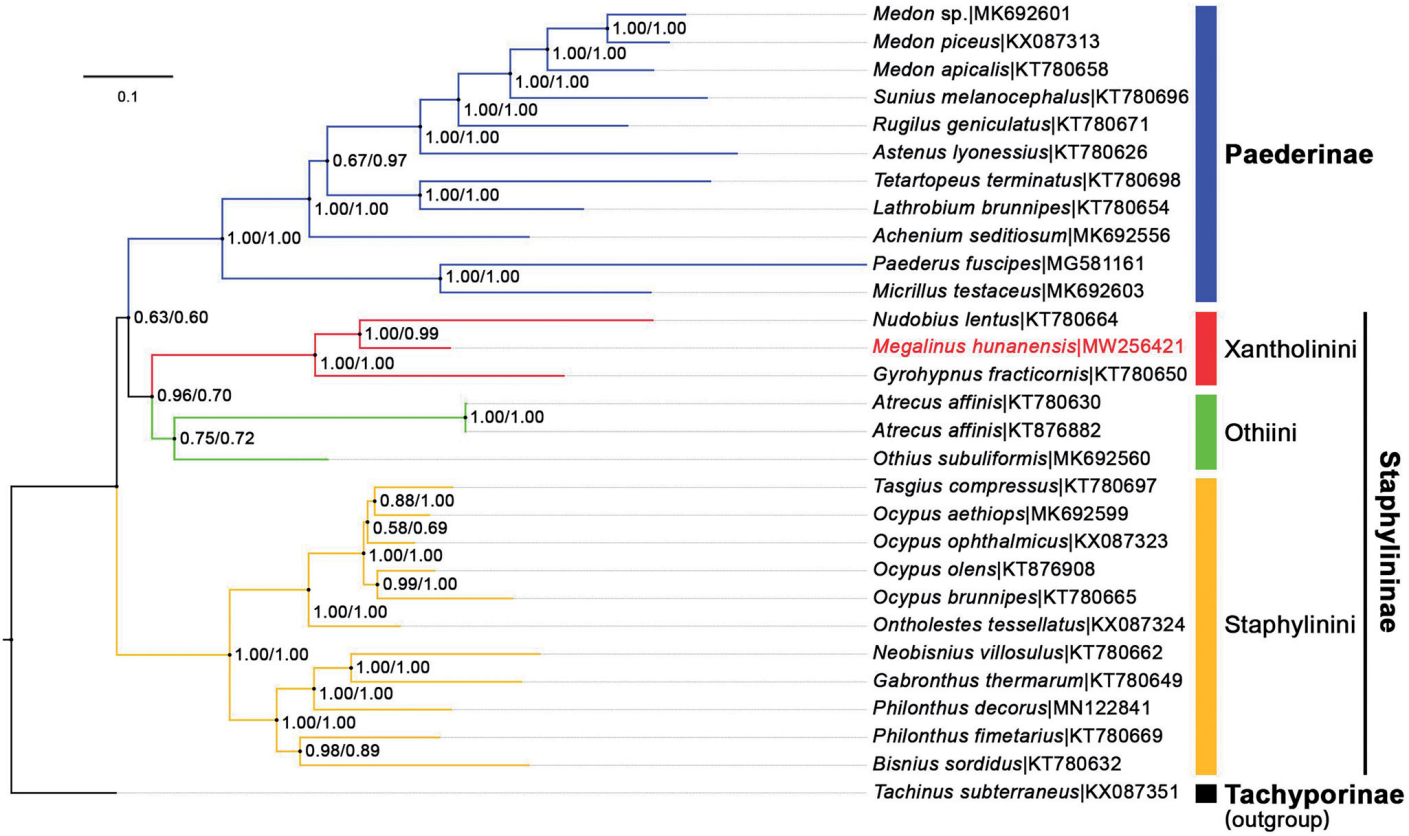
BI phylogenetic tree of the subfamilies Staphylininae + Paederinae, generated from the AA/DNA dataset (*M. hunanensis* nested in the tribe Xantholinini), both analyses based on different datasets showed the same topology. Bayesian posterior probabilities (BP Ps) were shown for each clade, and the GenBank accession numbers of all the species were listed in the tree.

## Data Availability

The genome sequence data that support the findings of this study are openly available in GenBank of NCBI at https://www.ncbi.nlm.nih.gov/nuccore/MW256421 under the accession no. MW256421. The associated BioProject, SRA, and Bio-Sample numbers are PRJNA732122, SRR14627589, and SAMN19312743, respectively.
